# Gas Particle Partitioning of PAHs Emissions from Typical Solid Fuel Combustions as Well as Their Health Risk Assessment in Rural Guanzhong Plain, China

**DOI:** 10.3390/toxics11010080

**Published:** 2023-01-15

**Authors:** Bin Zhang, Zezhi Peng, Jing Lv, Qin Peng, Kun He, Hongmei Xu, Jian Sun, Zhenxing Shen

**Affiliations:** Department of Environmental Science and Engineering, Xi’an Jiaotong University, Xi’an 710049, China

**Keywords:** gas and particle partitioning, benzo (a) pyrene toxicity equivalent, cancer risks, PAHs, residential solid fuel combustion

## Abstract

Air pollutants from the incomplete combustion of rural solid fuels are seriously harmful to both air quality and human health. To quantify the health effects of different fuel–stove combinations, gas and particle partitioning of twenty-nine species of polycyclic aromatic hydrocarbons (PAHs) emitted from seven fuel–stove combinations were examined in this study, and the benzo (a) pyrene toxicity equivalent (BaPeq) and cancer risks were estimated accordingly. The results showed that the gas phase PAHs (accounting for 68–78% of the total PAHs) had higher emission factors (EFs) than particulate ones. For all combustion combinations, pPAHs accounted for the highest proportion (84.5% to 99.3%) in both the gas and particulate phases, followed by aPAHs (0.63–14.7%), while the proportions of nPAHs and oPAHs were much lower (2–4 orders of magnitude) than pPAHs. For BaPeq, particulate phase PAHs dominated the BaPeq rather than gas ones, which may be due to the greater abundance of 5-ring particle PAHs. Gas and particle pPAHs were both predominant in the BaPeq, with proportions of 95.2–98.6% for all combustion combinations. Cancer risk results showed a descending order of bituminous coal combustion (0.003–0.05), biomass burning (0.002–0.01), and clean briquette coal combustion (10^−5^–0.001), indicating that local residents caused a severe health threat by solid fuel combustion (the threshold: 10^−4^). The results also highlighted that clean briquette coal could reduce cancer risks by 1–2 orders of magnitude compared to bulk coal and biomass. For oPAH, BcdPQ (6H-benzo(c,d)pyrene-6-one) had the highest cancer risk, ranging from 4.83 × 10^−5^ to 2.45 × 10^−4^, which were even higher than the total of aPAHs and nPAHs. The dramatically high toxicity and cancer risk of PAHs from solid fuel combustion strengthened the necessity and urgency of clean heating innovation in Guanzhong Plain and in similar places.

## 1. Introduction

Biomass and coal are typical solid fuels, which are the primary energy sources for rural residential heating and cooking [1]. Solid fuel combustion is generally deemed a main source of air pollution that affects not only developing regions but also some developed ones [2,3,4,5]. Residential solid fuel combustion has higher pollutant emission factors (EFs) due to the lower combustion efficiency compared with other combustion methods (such as industrial boilers) [6,7]. The low combustion efficiency and absence of emission control devices collectively result in residential solid fuels being the dominant contribution source of polycyclic aromatic hydrocarbons (PAHs) in the atmosphere [8].

PAHs have drawn much research interest due to their mutagenicity, genotoxicity, and carcinogenicity [9,10,11]. They were generally divided into different subgroups for various studies, including parent PAHs (pPAHs), alkylated PAHs (aPAHs), oxygenated PAHs (oPAHs), and nitrated PAHs (nPAHs). Among the monomers, Benzo(a)Pyrene (BaP) calls for most focus due to the highest known toxicity and is often used as the indicator of total PAHs. The adverse human health effects of PAHs depend on several factors, including the route and concentration of exposure, as well as the relative toxicity of PAHs [12]. Inhalation exposure of gas and particulate PAHs has been widely investigated, as they can easily facilitate their penetration into the respiratory organ and cause health adverse effects [13,14]. Until now, several studies have investigated atmospheric and indoor PAH levels and health risks. For example, Nadali et al. reported that the total PAH concentration ranged from 0.008 to 59.46 ng·m^−3^ and the average BaP equivalent carcinogenic (BaPeq) values in the cold season (averaging 0.35 ng·m^−3^) were higher than those in the warm season [15]. Xing et al. evaluated the human health risk of PM_2.5_-bound PAHs in Wuhan during the summer harvest season [16]. Cheng et al. investigated the BaPeq of eighteen particle phase PAHs emitted from different types of residential coal combustion [17]. In this study by Ma et al., a national-scale cancer risk assessment with atmospheric PAHs showed that BaPeq was 8.45 ± 14.1 ng/m^3^, which was higher than the new ambient air quality standards of China (GB 3095–2012, 1 ng/m^3^) [18]. Zhang et al. reported the emissions and their associated health risks of 16 priority-controlled PAHs in Jiangsu Province, and the results showed that the estimated cancer risks for different population groups were between 10^−6^ and 10^−5^, indicating high potential carcinogenic risks [19]. However, studies using BaPeq and cancer risks to directly assess the human health effects of PAHs from residential solid fuel combustion have rarely been reported, especially those covering both gas and particulate phase PAHs.

In the Guanzhong Plain of China, biomass fuels (e.g., branches, maize straw, and maize cob) and coals (including bulk bituminous coal and clean briquette coal) are widely used in the rural residential sector for cooking and heating usage. In this study, seven typical fuel–stove combinations in the rural area of Guanzhong Plain were selected for the investigation. Twenty-nine gas and particulate phase PAH monomers, including nineteen pPAHs, one aPAHs, one oPAHs, and eight nPAHs, were included in the examination. The objectives of the present study were: (1) to discuss the emission characteristics of gas and particulate phase PAHs from seven combinations, and (2) to investigate the BaPeq and cancer risks from two-phase PAH exposure. The results of this study are expected to better serve human health-oriented policy making and support the clean energy heating strategy in China.

## 2. Methods

### 2.1. Sample Collection and Chemical Analysis

This study was carried out in a rural village in Yijun County, Guanzhong Plain. Detailed fuel–stove groups can be found in Supplementary Text S1. Three parallel experiments were carried out for each group to ensure reproducibility. The completeness of all combustion could be determined by observing the burning status over the stove. The flame in the stove was extinguished, which evidenced that the fuel had been completely burned. Most particles were emitted from ignition to fierce combustion [20], and PM_2.5_ considered to be completely collected. Overall, the sampling had good representativeness. Each stove was connected with a flue gas chimney, and the diameter of the chimney and flue gas velocity were measured before sampling. The chimney diameter and the flue gas velocity of the two-stage and one-stage stoves were 13 cm and 0.7 m·s^−1^, respectively, and were 19 cm and 1.5 m·s^−1^ for the firewood stoves, respectively.

A self-made dilution sampling system was used to collected PM_2.5_ in flue emitted from the chimney, which was set next to the chimney exit on top of the roof, as shown in Supplementary Figure S1. The dilution tunnel was flow-controlled using multiple pumps to change the dilution ratio between 5 and 50 fold, as described in our previous publication [21]. Two quartz filters (Whatman, Maidstone, UK) and one Teflon filter (Pall Life Sciences, Ann Arbor, MI, USA) were used to collect PM_2.5_ for all tests using three mini-volume sample pumps (Airmetrics, Springfifield, OR, USA), and all tests were repeated at least three times. The flow rate was 5.0 L·min^−1^ which was calibrated by a primary flow calibrator (model 4140, TSI Incorporated, Shoreview, MN, USA) before each set of experiments. During the sampling, the particle-bound PAHs were first loaded onto the quartz filter membrane, and then gas phase PAHs were collected through two back-up PUF plugs. All quartz filters used in the tests were pre-heated at 900 °C for at least 5 h to remove residue impurities. Before being used, all filters were placed in air of 25 °C and 35% relative humidity for 48 h and then weighed by a microbalance with a sensitivity of ±1 µg (ME 5-F, Sartorius, Gottingen, Germany) [22]. The PUFs were properly wrapped with pre-baked aluminum foil. All samples were probably stored at −20 °C until the chemical analyses.

The analytical procedures were shown in our previous study and are briefly described here [23]. Twenty-nine PAHs were analyzed, including nineteen pPAHs and ten derivatives, as shown in Supplementary Table S1. The samples (quartz filter and PUF) were ultrasound extracted with 10% *v*/*v* diethyl ether in hexane for 16 h at 4 cycles per hour. After concentration and purification, the extract was analyzed with a trace gas chromatography-mass spectrometer (GC-MS) (7980GC/5975MS; Agilent Technology, Santa Clara, CA, USA). The detailed extraction procedures and instrumental setting are described in Supplementary Text S2. The abbreviation, limit of detection, quantified ion, and extraction recovery for each target analyte are summarized in Supplementary Tables S1 and S2. Quality assurance and quality control can be found in Supplementary Text S3.

### 2.2. Emission Factor Calculations and Indoor PAH Concentration Estimation

In this study, the mass-based PM_2.5_ and PAH emission factors (EFs) were defined as the mass emitted per unit mass fuel combusted (g·kg^−1^ or mg·kg^−1^) [24]. EFs were calculated following Equation (1):

(1) $$\mathrm{EFs}=\frac{m_{particle/PUF}\times DR\times S\times V}{Q_{filter}\times m_{fuel}}$$

where *m_particle/PUF_* is the mass of PM_2.5_ or PAHs deposited on the filter (mg) or stands for the gas phase PAHs mass determined for the entire PUF (µg or ng), DR is the dilution ratio during combustion sampling, *S* stands for cross sectional area of chimney (m^2^), *V* is the flow velocity of chimney with connect to stove (m/s), the stability of the velocity was monitored by real-time V-Trak flue gas anemometer (TSI Inc., Shoreview, MN, USA) and probe (Thermoanemometer Straight Probe 960, TSI Inc., Shoreview, MN, USA) throughout the combustion process, *Q_filter_* is the flow quantity of diluted smoke that pass through the filter (L·min^−1^), and *m_fuel_* is the mass of fuel combusted in each test (kg).

The indoor concentration estimation was conducted based on Equation (2):

(2) $$C_{indoor\;concentration}=\frac{m_{particle}\times DR\times DR_{1}}{Q_{filter}}$$

where *DR*_1_ refers to the dilution rate of PAHs from the combustion flue to the indoor environment. A similar method was used to estimate indoor pollution concentration [25]. The dilution ratio calculated using data in Sun et al. was 24.0 ± 22.2% [26]. The same ratio was used in the present study for indoor PAH concentration estimation.

### 2.3. Total BaPeq and Cancer Risk Estimation

The carcinogenic risk of a PAH mixture is often expressed by its BaPeq concentration [27]. The total BaPeq (TEQ) and MEQ (mutagenic equivalent) of gas and particulate phase PAHs were calculated based on Equations (3) and (4):

(3) $$\mathrm{TEQ}=C_{i}\times TEF_{i}$$

(4) $$\mathrm{MEQ}=C_{i}\times MEF_{i}$$

where *C_i_* is the concentration of PAH congener *i*; $TEF_{i}$ is the toxicity equivalency factor (TEF) of PAH congener *i;* $MEF_{i}$ is the mutagenic equivalency factor (TEF) of PAH congener *I;* All TEF and MEF are summed in Supplementary Table S3.

Cancer risks (CR) were estimated according to Equation (5):

(5) CR=SF×C×IR×ET×ED/(BW×AL×NY)

where *SF* is the cancer slope factor for BaP and is set to 3.14 × 10^−3^ (kg·day·µg^−1^), *C* is the total TEQ concentration for PAHs in indoor environment (µg·m^−3^), *IR* refers to the inhalation rate (m^3^·h^−1^), *ET* refers to the exposure duration (h·day^−1^), *ED* is the total number of exposure days, *BW* stands for the body weight (kg), *AL* is the average lifetime (year), *NY* is the total number of days in 1 year (365 day·year^−1^). All parameters were obtained from Sun et al., as shown in Supplementary Table S4 [28].

## 3. Results and Discussion

### 3.1. Gas Particle Partitioning of PAHs

The means and standard deviation (SD) of the EFs of pPAHs, aPAHs, oPAHs, and nPAHs are listed in Table 1. The results implied that the gas and particulate phases of PAH EFs from the two-stage stove were 1.1–2.4 times higher than those of the one-stage stove. In contrast to some earlier studies, this study observed that using two-stage stoves decreased heat transfer efficiency and increased pollutant emissions compared to one-stage stoves (old-fashioned stoves) [29,30,31]. This was potentially attributed to the fact that the secondary air supply for the two-stage stove was insufficient to achieve optimum combustion efficiency due to improper installation. Sun et al. reported that secondary air supply volume has a quadric effect on combustion efficiency [32]. Either insufficient or excessive secondary air would reduce combustion efficiencies, resulting in more pollutant emissions. Meanwhile, fuel types also highly affected PAH emissions; that is, higher gas and particulate phases PAH EFs were seen for bituminous coal (the average EFs of gas and particulate phases PAHs were 714 ± 382 mg·kg^−1^ and 220 ± 62.8 mg·kg^−1^, respectively) than clean briquette coal combustion (the average EFs of gas and particulate phases PAHs were 15.3 ± 7.45 mg·kg^−1^ and 5.26 ± 3.19 mg·kg^−1^, respectively) regardless of stove types. The results indicated that clean briquette coal was efficient in reducing the emissions of PAHs. This may be attributed to the higher combustion efficiency of clean briquette coal compared to bituminous coal [33].

For the biomass fuels, the highest EFs of gas and particulate phase PAHs were observed for the maize straw (643 ± 276 mg·kg^−1^ and 302 ± 144 mg·kg^−1^, respectively), followed by the wood branch (557 ± 31.0 mg·kg^−1^ and 234 ± 2.35 mg·kg^−1^, respectively), and the maize cob (471 ± 55.4 mg·kg^−1^ and 196 ± 6.53 mg·kg^−1^, respectively). However, the EFs of PAHs from biomass burning were 1–2 orders of magnitude higher than those of clean briquette coal, implying that clean briquette coal replacing biomass was an effective pathway to reduce pollutant emissions. As shown in Table 1, gas phase PAHs exceeded 2/3 of the total PAH emissions due to naphthalene (NAP) mostly existed in the gas phase [23]. In addition, for all combinations, pPAHs accounted for the highest proportion (84.5% to 99.3%) in both the gas and particulate phases due to the fact that pPAHs were mainly derived from primary emissions, while derivatives were mostly produced during atmospheric aging. Among the derivatives, the proportions of nPAHs and oPAHs were negligible, which were 2–4 orders of magnitude lower than those of pPAHs. As reported by Keyte et al., pPAHs reacted with radicals to generate energy-rich intermediate products that would further react with NO_2_ or O_2_ to yield nPAHs and oPAHs, respectively [34].

### 3.2. BaPeq

**Figure 1:**
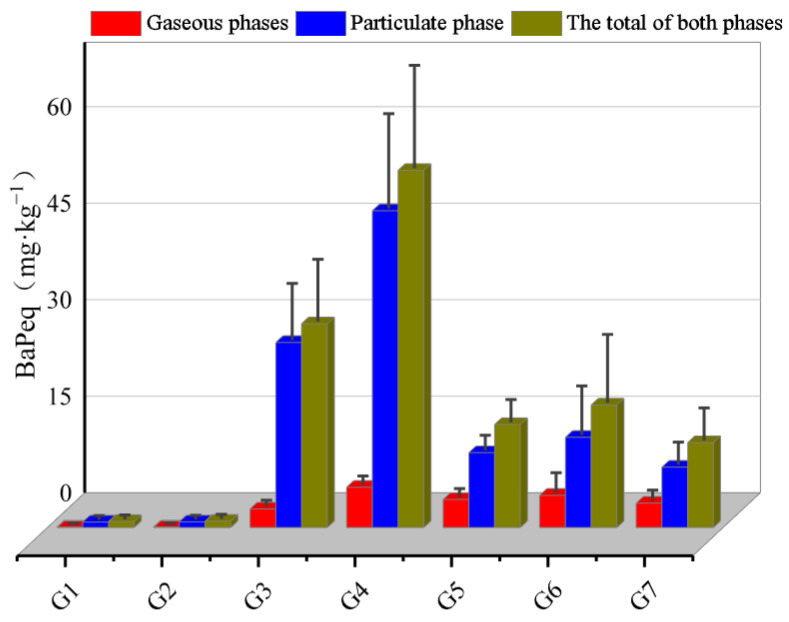
The gas and particulate phases BaPeq of seven fuel–stove combinations.

To assess the potential risks of PAHs under different combustion scenarios, the total gas and particulate phases BaPeq and MEQ of seven combinations were calculated, and the results are shown in Figure 1 and Supplementary Table S5. The results showed that bituminous coal combustion had higher mutagenic values than other combustion scenarios. BaPeq for the seven solid fuel combustion scenarios ranged from 0.95 to 55.5 mg·kg^−1^, which were comparable with those observed by Ngo et al. (34.4 ± 23.9 mg·kg^−1^) and Zhang et al. (2.79–41.9 mg·kg^−1^) [10,35]. BaPeq was highly fuel-type dependent; that was, the average BaPeq followed the decreasing order of bituminous coal (43.6 mg·kg^−1^) > biomass (16.1 mg·kg^−1^) > clean briquette coal (0.97 mg·kg^−1^), which was the same as PAH EFs. The extremely low BaPeq from clean briquette coal combustion indicated that clean briquette coal technology was conducive to reducing the emission of carcinogenic PAHs; similar results had been widely reported as well by Li et al. and Xu et al. [36,37]. In addition, it was found that the BaPeq values and PAH EFs ranked differently among the scenarios, suggesting that the EFs of PAHs did not effectively reflect their carcinogenicity. This result indicated that human health-oriented emission assessment was more meaningful than total pollutant emission assessment [38]. According to Figure 1, particulate phase PAHs accounted for less than one-third of the total PAH emissions but contributed 71.1% to 91.7% of the total BaPeq, indicating that gas phase PAHs were relatively less carcinogenic, while particulate phase PAHs were the dominant. This result might be attributed to the fact that the high molecular weight PAHs (4-, 5-, and 6-ring ones) were dominantly distributed in the particulate phase, which had high toxicity but rarely existed in the gas phase [1,39,40].

**Figure 2:**
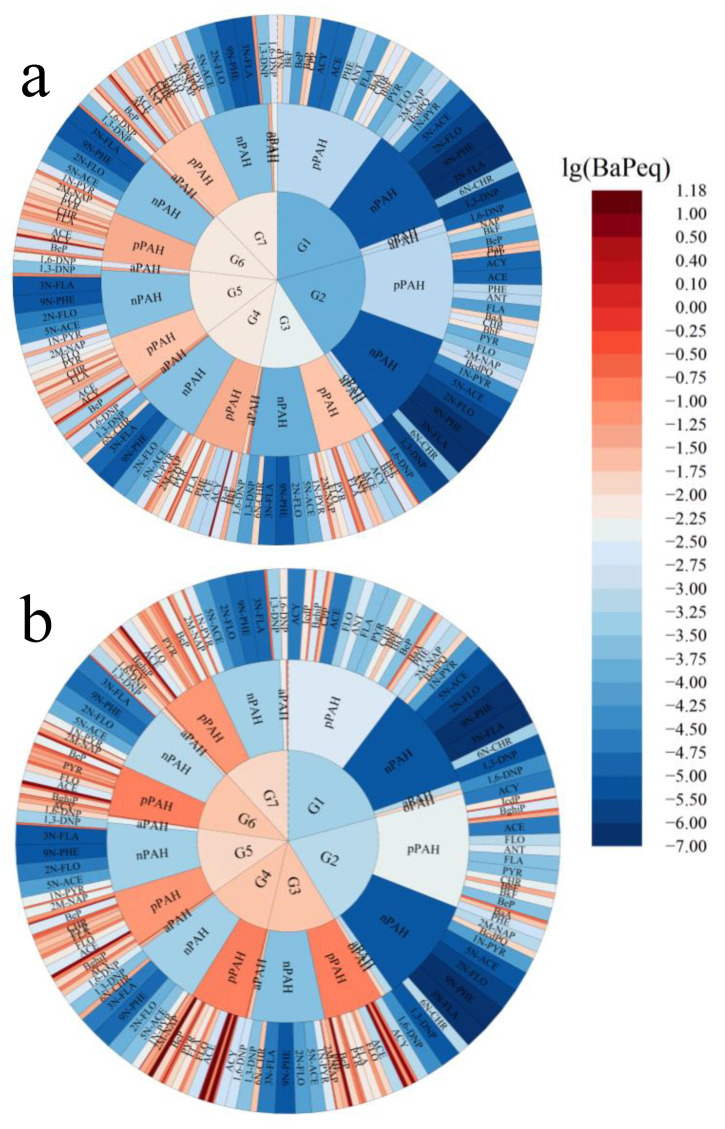
The contribution of four subgroups and individual species PAHs obtained from seven fuel–stove combinations combustion to total BaPeq ((**a**) refers to gas phase BaPeq, (**b**) refers to particulate phase PAHs).

Figure 2 reflects the contributions of four subgroups and individual species PAHs obtained from seven scenarios to the total BaPeq. pPAHs was the absolute dominant source of BaPeq regardless of gas and particulate phases, accounting for over 95% averagely in all scenarios. This result might lead to a lack of research on derivatives, and further studies are needed. For coal combustion scenarios, the dominant contributors within the total BaPeq of gas phase pPAHs were BaP and cyclopenta [cd] pyrene (CPP), accounting for 20.5% to 44.2%. However, the dominant contributors within the total BaPeq of particulate phase pPAHs were dibenzo[a,h]anthracene (DBahA) and dibenzo[a,e]pyrene (DBaeP), accounting for 25.9% to 38.1%. For biomass burning scenarios, BaPeq from CPP was the highest contributor within the total BaPeq of gas and particulate phase pPAHs, contributing 66.1% to 74.0% and 56.2% to 62.3%, respectively. These differences were related to their respective emissions characteristics. The contributions of aPAHs, oPAHs, and nPAHs to the total BaPeq were one to three orders of magnitude lower than those of the pPAHs. Although only one oPAH species (6H-benzo(c,d)pyrene-6-one) was included in the BaPeq calculation, it ranked 4-7th among all monomers, emphasizing the significant role that oPAHs played in equivalent toxic estimation. Considering that many oPAHs did not yet have toxicity equivalence factors, the health effects of oPAHs might be further underestimated.

### 3.3. Contribution of Different Ring PAHs to BaPeq

Generally, PAHs could be classified into five groups based on the number of aromatic rings in the structure of the PAHs molecule: 2-rings (Nap, ACY, ACE, FLO, 2M-NAP, 5N-ACE, and 2N-FLO), 3-rings (PHE, ANT, FLA, 9N-PHE, and 3N-FLA), 4-rings (PYR, BaA, CHR, BbF, BkF, CPP, 1N-PYR, 6N-CHR, 1,3-DNP, and 1,6-DNP), 5-rings (BaP, BeP, IcdP, DBahA, and BcdPQ), and 6-rings (BghiP and DBaeP) [41]. In this study, the total PAHs were further classified into low (2- and 3-ring), medium (4-ring), and high molecular weights (5- and 6-ring) (LMW, MMW, and HMW as abbreviations, respectively).

**Figure 3:**
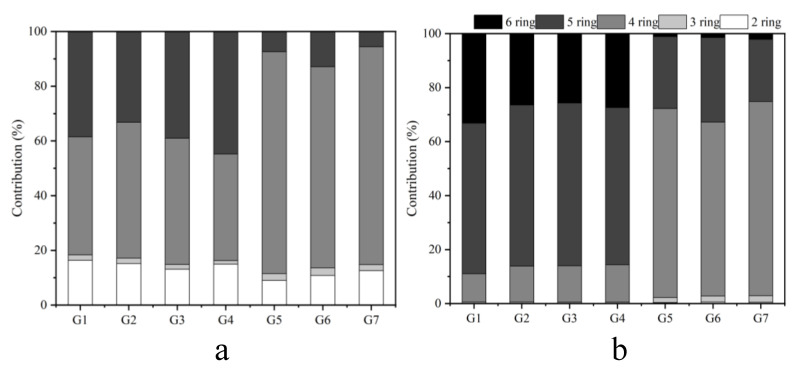
The contribution of PAHs with different ring numbers to the total BaPeq ((**a**) refers to the gas phase, (**b**) refers to the particulate phase).

Supplementary Figure S2 illustrates the distributions of PAH EFs based on the number of aromatic rings. In the gas phase, the proportion of 2-ring PAHs emitted from the coal and biomass was the largest, accounting for 84% to 94%. In the particulate phase, 3-ring PAHs was the largest contribution source, accounting for 32% to 59%. Figure 3 presents the contribution of PAHs with different ring numbers to the total BaPeq. The ring distributions of PAH EFs and BaPeq had dramatic differences. For BaPeq of gas phase PAHs, 4-ring PAHs was the largest contributor for all scenarios except G4, accounting for 43.1% to 81.1% of total gaseous BaPeq. Followed by 5-ring PAHs (5.61–44.8%), 2-ring PAHs (9.02–16.4%), and 3-ring PAHs (1.28–2.78%). For particulate phase BaPeq, 5-ring PAHs instead of 4-ring ones in the gas phase became the largest contributor for coal combustion scenarios, accounting for over 55%, while 4-ring PAHs was still the dominant contributor for biomass burning scenarios, accounting for 64.4% to 71.9% in particulate phase BaPeq. Regardless of both phases, the contribution of 2-ring PAHs to BaPeq was the lowest for all combinations, ranging from 0.14% to 0.55%, because of the relatively low toxicity of LMW PAHs. As reported by Ray et al., HMW PAHs had high toxicity due to their low water solubility, lipophilicity, and high stability [42]. The proportion of HMW PAHs in bituminous coal was higher than that in biomass, which explained the high BaPeq of bituminous coal.

### 3.4. Cancer Risk Assessment

Due to the mutagenicity, genotoxicity, and carcinogenicity of PAHs, the health risk assessment of PAHs has been widely employed in the literature [9,10,43,44]. Based on the variant PAH emissions from different combustion scenarios, PAH exposure-related cancer risks are assessed below.

**Figure 4:**
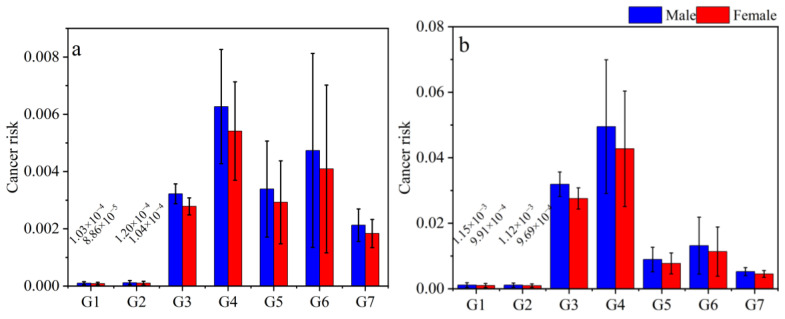
Cancer risks estimated from indoor gas and particulate phases PAHs exposures ((**a**) refers to gas phase, (**b**) refers to particulate phase).

Cancer risk estimations from indoor gas and particulate phase PAH exposures are presented in Figure 4. According to the reference U.S. EPA (1980), a one-in-a-million chance of additional human cancer over a 70-year lifetime (Cancer Risk = 10^−6^) is the level of risk considered acceptable or inconsequential, whereas an additional lifetime cancer risk of one in ten thousand or greater (Cancer Risk ≥ 10^−4^) is considered serious, and there is a high priority for paying attention to such health problems. For example, the mean cancer risk value was reported to be 3.85 × 10^−5^ to 4.36 × 10^−5^ for population in the study by Mosallaei et al., indicating potential cancer risk as a result of exposure to PAHs [9]. Liu et al. reported that the average values of the sum of cancer risks were 2.22 × 10^−7^ for adults and 2.51 × 10^−7^ for children, suggesting that there is a low health risk posed by PAHs [45]. According to Figure 4, the descending order of cancer risks caused by different fuel combustions were bituminous coal combustion > biomass burning > clean briquette coal combustion for males and females (there was no significant difference in cancer risks between males and females in the present study). The cancer risks of particulate phase PAHs were one order of magnitude higher than those of gas phase PAHs, implying that particulate phase PAH exposure resulted in much more cancer risks than gas phase ones. For both phases, significant reductions (*p* < 0.05) of cancer risks were found for clean briquette coal combustion scenarios compared to bituminous coal and biomass burning scenarios. Although the reduction could not turn the cancer risks to a safe level (i.e., <1 × 10^−6^), it did reduce cancer risks by one to two orders of magnitude. As discussed in Section 3.1, a change from a one-stage stove to a two-stage stove led to an increase in cancer risks, from 3 × 10^−3^ to 5.84 × 10^−3^ and 2.98 × 10^−2^ to 4.62 × 10^−2^ for gas and particulate phases, respectively. It showed that the use of a two-stage stove did not necessarily achieve the goal of reducing cancer risks and required scientific installation and proper use to ensure optimal effects.

Supplementary Tables S6–S9 exhibit the cancer risks caused by individual gas and particulate phases PAH species exposure for both genders. Most of the cancer risks were still caused by pPAHs due to their high EFs and concentrations. Because of the lack of attention to oPAHs in previous studies [27,46], BcdPQ was the only species from all oPAHs detected in this study that had TEQ values [43]. Even in this case, the cancer risk of BcdPQ remained as high as 4.83 × 10^−5^ to 2.45 × 10^−4^. As a monomer, it ranked 4–7th in cancer risks, not only higher than most pPAHs but also higher than the sum of aPAHs and nPAHs cancer risks (the average values were 6.69 × 10^−6^ to 1.80 × 10^−5^). Therefore, oPAHs need more research and attention.

## 4. Conclusions

This study measured the EFs of 29 gas and particulate phase PAHs from seven fuel–stove combinations and evaluated their BaPeq and cancer risks. It was found that particulate phase PAHs dominated the total BaPeq (over 71.1%) with less than 33.3% of total PAH emissions. Cancer risk exposure to PAHs from all combustion combinations was over the threshold (10^−4^), emphasizing the strong health threat to residents caused by solid fuel combustion. For individual PAH monomers, BcdPQ (the sole monomer of oPAHs) exhibited comparably high cancer risks as major pPAHs and was even higher than the sum of aPAHs and nPAHs, revealing the significance and adverse effects of oPAHs on human health. This study highlighted the non-negligible toxicity and cancer risk of PAHs from solid fuel combustion and demonstrated the effectiveness of clean heating measures in mitigating these toxicity and health risks.

## Figures and Tables

**Table 1:** Summary of the EF_pPAH_, EF_aPAH_, EF_oPAH_, and EF_nPAH_ for the seven fuel–stove combustions (mean ± standard deviation).

Group	Gas Phase PAHs (mg/kg)					Particulate Phase PAHs (mg/kg)					
	pPAH	aPAH	oPAH	nPAH	∑PAHs	pPAH	aPAH	oPAH	nPAH	∑PAHs	Total PAHs
G1	13.4 ± 4.73	0.63 ± 0.54	0.01 ± 0.01	0.002 ± 0.00	14.1 ± 5.29	4.25 ± 2.60	0.73 ± 0.64	0.04 ± 0.02	0.002 ± 0.00	5.03 ± 3.26	19.1 ± 8.55
G2	15.8 ± 9.41	0.58 ± 0.19	0.01 ± 0.01	0.001 ± 0.00	16.4 ± 9.61	4.81 ± 2.71	0.63 ± 0.39	0.04 ± 0.01	0.002 ± 0.00	5.48 ± 3.11	21.9 ± 12.7
G3	407 ± 46.7	17.7 ± 7.21	0.34 ± 0.18	0.05 ± 0.01	425 ± 54.1	140 ± 32.4	24.2 ± 10.4	1.32 ± 0.55	0.08 ± 0.02	165 ± 43.4	590 ± 97.5
G4	970 ± 306	32.7 ± 22.0	0.32 ± 0.05	0.06 ± 0.03	1003 ± 328	237 ± 63.1	36.3 ± 18.2	1.97 ± 0.82	0.13 ± 0.06	275 ± 82.2	1278 ± 410
G5	468 ± 53.6	2.96 ± 1.81	0.15 ± 0.06	0.06 ± 0.00	471 ± 55.4	191 ± 4.91	3.86 ± 1.38	0.97 ± 0.25	0.13 ± 0.01	196 ± 6.53	667 ± 61.9
G6	629 ± 273	13.3 ± 2.97	0.32 ± 0.14	0.09 ± 0.04	643 ± 276	284 ± 134	15.5 ± 8.60	1.74 ± 1.09	0.18 ± 0.09	302 ± 144	945 ± 420
G7	553 ± 32.5	3.61 ± 1.67	0.10 ± 0.15	0.05 ± 0.01	557 ± 31.0	230 ± 1.75	3.66 ± 0.31	0.63 ± 0.89	0.11 ± 0.01	234 ± 2.35	791 ± 33.4

## Data Availability

Not applicable.

## Supplementary Materials

The following supporting information can be downloaded at: https://www.mdpi.com/article/10.3390/toxics11010080/s1, Figure S1: The simple structure of dilution system; Figure S2: The number of aromatic rings distributions of PAHs in gaseous (a) and particulate phases (b); Table S1: Individual profile of PAHs (MW = molar weight, LOD = limit of detection) (Mean ± Standard Deviation); Table S2: The quantified ion and extraction recovery for each PAHs (SD = Standard Deviation); Table S3: TEF of 29 PAHs involved in this study; Table S4: Parameters settings in non-cancer and cancer risk assessment; Table S5: BaP_MEQ_ (MEQ) of gas and particulate phases PAHs from seven different combustion scenarios; Table S6: Cancer risk for Males of individual PAHs species in gaseous phase; Table S7: Cancer risk for Females of individual PAHs species in gaseous phase; Table S8: Cancer risk for Males of individual PAHs species in particulate phase; Table S9: Cancer risk for Females of individual PAHs species in particulate phase.

## References

[1] Tao S., Ru M.Y., Du W., Zhu X., Zhong Q.R., Li B.G., Shen G.F., Pan X.L., Meng W.J., Chen Y.L. (2018). Quantifying the rural residential energy transition in China from 1992 to 2012 through a representative national survey. Nat. Energy.

[2] Lin C., Huang R.-J., Ceburnis D., Buckley P., Preissler J., Wenger J., Rinaldi M., Facchini M.C., O’Dowd C., Ovadnevaite J. (2018). Extreme air pollution from residential solid fuel burning. Nat. Sustain..

[3] Shen Z., Arimoto R., Cao J., Zhang R., Li X., Du N., Okuda T., Nakao S., Tanaka S. (2008). Seasonal Variations and Evidence for the Effectiveness of Pollution Controls on Water-Soluble Inorganic Species in Total Suspended Particulates and Fine Particulate Matter from Xi’an, China. J. Air Waste Manag. Assoc..

[4] Shen Z., Cao J., Arimoto R., Han Z., Zhang R., Han Y., Liu S., Okuda T., Nakao S., Tanaka S. (2009). Ionic composition of TSP and PM_2.5_ during dust storms and air pollution episodes at Xi’an, China. Atmos. Environ..

[5] Wang X., Shen Z., Liu F., Lu D., Tao J., Lei Y., Zhang Q., Zeng Y., Xu H., Wu Y. (2018). Saccharides in summer and winter PM_2.5_ over Xi’an, Northwestern China: Sources, and yearly variations of biomass burning contribution to PM_2.5_. Atmos. Res..

[6] Shen G., Tao S., Wei S., Chen Y., Zhang Y., Shen H., Huang Y., Zhu D., Yuan C., Wang H. (2013). Field Measurement of Emission Factors of PM, EC, OC, Parent, Nitro-, and Oxy- Polycyclic Aromatic Hydrocarbons for Residential Briquette, Coal Cake, and Wood in Rural Shanxi, China. Environ. Sci. Technol..

[7] Zhang Y., Schauer J.J., Zhang Y., Zeng L., Wei Y., Liu Y., Shao M. (2008). Characteristics of Particulate Carbon Emissions from Real-World Chinese Coal Combustion. Environ. Sci. Technol..

[8] Mastral A.M., Callén M.S. (2000). A Review on Polycyclic Aromatic Hydrocarbon (PAH) Emissions from Energy Generation. Environ. Sci. Technol..

[9] Mosallaei S., Hashemi H., Hoseini M., Dehghani M., Naz A. (2023). Polycyclic Aromatic Hydro-carbons (PAHs) in household dust: The association between PAHs, Cancer Risk and Sick Building Syndrome. Build. Environ..

[10] Ngo T.H., Yang H.Y., Pan S.Y., Chang M.B., Chi K.H. (2021). Condensable and filterable particulate matter emission of coal fired boilers and characteristics of PM_2.5_-bound polycyclic aromatic hydrocarbons in the vicinity. Fuel.

[11] Wincent E., Le Bihanic F., Dreij K. (2016). Induction and inhibition of human cytochrome P4501 by oxygenated polycyclic aromatic hydrocarbons. Toxicol. Res..

[12] Škrbić B., Đurišić-Mladenović N., Živančev J., Tadić Đ. (2018). Seasonal occurrence and cancer risk assessment of polycyclic aromatic hydrocarbons in street dust from the Novi Sad city, Serbia. Sci. Total. Environ..

[13] Chen Y.-C., Chiang H.-C., Hsu C.-Y., Yang T.-T., Lin T.-Y., Chen M.-J., Chen N.-T., Wu Y.-S. (2016). Ambient PM_2.5_-bound polycyclic aromatic hydrocarbons (PAHs) in Changhua County, central Taiwan: Seasonal variation, source apportionment and cancer risk assessment. Environ. Pollut..

[14] Hoseini M., Yunesian M., Nabizadeh R., Yaghmaeian K., Ahmadkhaniha R., Rastkari N., Parmy S., Faridi S., Rafiee A., Naddafi K. (2015). Characterization and risk assessment of polycyclic aromatic hydrocarbons (PAHs) in urban atmospheric Particulate of Tehran, Iran. Environ. Sci. Pollut. Res..

[15] Nadali A., Leili M., Bahrami A., Karami M., Afkhami A. (2021). Phase distribution and risk assessment of PAHs in ambient air of Hamadan, Iran. Ecotoxicol. Environ. Saf..

[16] Xing X., Chen Z., Tian Q., Mao Y., Liu W., Shi M., Cheng C., Hu T., Zhu G., Li Y. (2020). Characterization and source identification of PM_2.5_-bound polycyclic aromatic hydrocarbons in urban, suburban, and rural ambient air, central China during summer harvest. Ecotoxicol. Environ. Saf..

[17] Cheng Y., Kong S., Yan Q., Liu H., Wang W., Chen K., Yin Y., Zheng H., Wu J., Yao L. (2019). Size-segregated emission fac-tors and health risks of PAHs from residential coal flaming/smoldering combustion. Environ. Sci. Pollut. Res..

[18] Ma W.-L., Zhu F.-J., Liu L.-Y., Jia H.-L., Yang M., Li Y.-F. (2020). PAHs in Chinese atmosphere Part II: Health risk assessment. Ecotoxicol. Environ. Saf..

[19] Zhang P., Zhou Y., Chen Y., Yu M., Xia Z. (2022). Construction of an atmospheric PAH emission inventory and health risk assessment in Jiangsu, China. Air Qual. Atmos. Health.

[20] Zhou W., Jiang J., Duan L., Hao J. (2016). Evolution of Submicrometer Organic Aero-sols during a Complete Residential Coal Combustion Process. Environ. Sci. Technol..

[21] Sun J., Shen Z., Zhang L., Zhang Y., Zhang T., Lei Y., Niu X., Zhang Q., Dang W., Han W. (2019). Volatile organic compounds emissions from traditional and clean domestic heating appliances in Guanzhong Plain, China: Emission factors, source profiles, and effects on regional air quality. Environ. Int..

[22] Shen Z.X., Cao J.J., Arimoto R., Zhang R.J., Jie D.M., Liu S.X., Zhu C.S. (2007). Chemical composition and source characterization of spring aerosol over Horqin sand land in northeastern China. J. Geophys. Res. Atmos..

[23] Zhang B., Sun J., Jiang N., Zeng Y., Zhang Y., He K., Xu H., Liu S., Ho S.S.H., Qu L. (2021). Emission factors, characteristics, and gas-particle partitioning of polycyclic aromatic hydrocarbons in PM_2.5_ emitted for the typical solid fuel combustions in rural Guanzhong Plain, China. Environ. Pollut..

[24] Delmas R., Lacaux J.P., Brocard D. (1995). Determination of biomass burning emission factors: Methods and results. Environ. Monit. Assess..

[25] Zhang Y., Shen Z., Sun J., Zhang L., Zhang B., Zhang T., Wang J., Xu H., Liu P., Zhang N. (2020). Parent, alkylated, oxygenated and nitro polycyclic aromatic hydrocarbons from raw coal chunks and clean coal combustion: Emission factors, source profiles, and health risks. Sci. Total. Environ..

[26] Sun J., Wang J., Shen Z., Huang Y., Zhang Y., Niu X., Cao J., Zhang Q., Xu H., Zhang N. (2019). Volatile organic compounds from residential solid fuel burning in Guanzhong Plain, China: Source-related profiles and risks. Chemosphere.

[27] Bandowe B.A.M., Meusel H., Huang R.-J., Ho K., Cao J., Hoffmann T., Wilcke W. (2014). PM_2.5_-bound oxygenated PAHs, nitro-PAHs and parent-PAHs from the atmosphere of a Chinese meg-acity: Seasonal variation, sources and cancer risk assessment. Sci. Total Environ..

[28] Sun J., Shen Z., Zhang T., Kong S., Zhang H., Niu X., Huang S., Xu H., Ho K.-F., Cao J. (2022). A comprehensive evaluation of PM_2.5_-bound PAHs and their derivative in winter from six megacities in China: Insight the source-dependent health risk and secondary reactions. Environ. Int..

[29] MacCarty N., Still D., Ogle D. (2010). Fuel use and emissions performance of fifty cooking stoves in the laboratory and related benchmarks of performance. Energy Sustain. Dev..

[30] Manoj K., Sachin K., Tyagi S.K. (2013). Design, development and technological advancement in the biomass cookstoves: A review. Renew. Sustain. Energy Rev..

[31] Ohura T., Kurihara R., Hashimoto S. (2010). Aryl hydrocarbon receptor activities of hydroxylated polycyclic aromatic hydrocarbons in recombinant yeast cells. Toxicol. Environ. Chem..

[32] Sun J., Shen Z., Zhang L., Zhang Q., Lei Y., Cao J., Huang Y., Liu S., Zheng C., Xu H. (2017). Impact of primary and secondary air supply intensity in stove on emissions of size-segregated particulate matter and carbonaceous aerosols from apple tree wood burning. Atmos. Res..

[33] Zhang Y., Shen Z., Zhang B., Sun J., Zhang L., Zhang T., Xu H., Bei N., Tian J., Wang Q. (2019). Emission reduction effect on PM_2.5_, SO_2_ and NO_x_ by using red mud as additive in clean coal briquetting. Atmos. Environ..

[34] Keyte I.J., Harrison R.M., Lammel G. (2013). Chemical reactivity and long-range transport potential of polycyclic aromatic hydrocarbons—A review. Chem. Soc. Rev..

[35] Zhang Y., Shen Z., Sun J., Zhang L., Zhang B., Zou H., Zhang T., Ho S.S.H., Chang X., Xu H. (2020). Parent, alkylated, oxygenated and nitrated polycyclic aromatic hydrocarbons in PM_2.5_ emitted from residential biomass burning and coal combustion: A novel database of 14 heating scenarios. Environ. Pollut..

[36] Li Y., Xu H., Wang J., Ho S.S.H., He K., Shen Z., Ning Z., Sun J., Li L., Lei R. (2019). Personal exposure to PM_2.5_-bound organic species from domestic solid fuel combustion in rural Guanzhong Basin, China: Characteristics and health implication. Chemosphere.

[37] Xu H., Li Y., Guinot B., Wang J., He K., Ho K.F., Cao J., Shen Z., Sun J., Lei Y. (2018). Personal exposure of PM_2.5_ emitted from solid fuels combustion for household heating and cooking in rural Guanzhong Plain, northwestern China. Atmos. Environ..

[38] Sun J., Niu X., Zhang B., Zhang L., Yu J., He K., Zhang T., Wang Q., Xu H., Cao J. (2022). Clarifying winter clean heating importance: Insight chemical compositions and cytotoxicity exposure to primary and aged pollution emissions in China rural areas. J. Environ. Manag..

[39] Feng S., Shen X., Hao X., Cao X., Li X., Yao X., Shi Y., Lv T., Yao Z. (2021). Polycyclic and ni-tro-polycyclic aromatic hydrocarbon pollution characteristics and carcinogenic risk assessment of in-door kitchen air during cooking periods in rural households in North China. Environ. Sci. Pollut. Res..

[40] Liu H.-H., Yang H.-H., Chou C.-D., Lin M.-H., Chen H.-L. (2010). Risk assessment of gaseous/particulate phase PAH exposure in foundry industry. J. Hazard. Mater..

[41] Chen P., Kang S., Li C., Li Q., Yan F., Guo J., Ji Z., Zhang Q., Hu Z., Tripathee L. (2018). Source Apportionment and Risk Assessment of Atmospheric Polycyclic Aromatic Hydrocarbons in Lhasa, Tibet, China. Aerosol Air Qual. Res..

[42] Ray D., Ghosh S.K., Raha S. (2018). Impacts of photochemical ageing on the half-lives and diagnostic ratio of polycyclic aromatic hydrocarbons intrinsic to PM_2.5_ collected from ‘real-world’ like combustion events of wood and rice straw burning. J. Hazard. Mater..

[43] Durant J.L., Busby W.F., Lafleur A.L., Penman B.W., Crespi C.L. (1996). Human cell mutagenicity of oxygenated, nitrated and unsubstituted polycyclic aromatic hydrocarbons associated with urban aerosols. Mutat. Res. Genet. Toxicol..

[44] Lui K., Bandowe B.A.M., Tian L., Chan C.-S., Cao J.-J., Ning Z., Lee S., Ho K. (2017). Cancer risk from polycyclic aromatic compounds in fine particulate matter generated from household coal combustion in Xuanwei, China. Chemosphere.

[45] Liu B., Huang F., Yu Y., Dong W. (2021). Polycyclic Aromatic Hydrocarbons (PAHs) in Indoor Dust Across China: Occurrence, Sources and Cancer Risk Assessment. Arch. Environ. Contam. Toxicol..

[46] Wei C., Han Y., Bandowe B.A.M., Cao J., Huang R.-J., Ni H., Tian J., Wilcke W. (2015). Occurrence, gas/particle partitioning and carcinogenic risk of polycyclic aromatic hydrocarbons and their oxygen and nitrogen containing derivatives in Xi’an, central China. Sci. Total. Environ..

