# Supplementary material for: Gas Particle Partitioning of PAHs Emissions from Typical Solid Fuel Combustions as Well as Their Health Risk Assessment in Rural Guanzhong Plain, China
Source: Toxics. 2023 Jan 15;11(1):80. doi: 10.3390/toxics11010080 (PMC9863936; doi:10.3390/toxics11010080)
Supplement: Supplementary file 1 [file toxics-11-00080-s001.zip › toxics-2139451-supplementary.pdf]

toxics

Supplementary Materials:

Gas Particle Partitioning of PAHs Emissions from Typical Solid Fuel Combustions as Well as Their Health Risk Assessment in Rural Guanzhong Plain, China

Text S1

According to the ubiquities of fuel and stove in the region, five types of solid fuels (i.e. clean briquette coal, bituminous coal, maize cob, maize stalk, and wood branch) and three stoves (i.e. one-stage stove, two-stage stove, and firewood stove) were made up into seven fuel-stove groups, which were clean briquette coal and one-stage stove (G1), clean briquette coal and two-stage stove (G2), bituminous coal and one-stage stove (G3), bituminous coal and two-stage stove (G4), maize cob and firewood stove (G5), maize straw and firewood stove (G6), wood branch and firewood stove (G7), respectively. All combustion experiments were conducted strictly in accordance with the habits of the residents.

Text S2

The PAHs in the samples were extracted by using high purity grade solvents. Each sampling media, PUF or quartz filter, was extracted with 10% v/v diethyl ether in hexane for 16 h at 4 cycles per hour using Soxhlet extractor. Each extract was evaporated to dryness, recombined with cyclohexane and concentrated in kuderna Danish concentrator tube. The extract was then cleaned up with a disposable silica gel column, eluted by using 40% v/v dichloromethane in pentane. The eluent was evaporated and reconstituted with 1 ml acetonitrile. The concentrated extract was vortex mixed and transferred to a brown glass bottle for chromatographic analysis. The final solution was then mixed with 25 mL injection internal standard (fluoranthene-d10, 20 ng/mL) to a total volume of 1 ml. One microliter of each extract was injected into a GC system (7890N, Agilent Technology, Santa Clara, CA) at 280 °C with an autosampler (7693, Agilent Technology). The operating temperature of the injector was set at 250 °C in a splitless mode. The GC oven temperature was initially set at 50 °C, ramped to 150 °C in 4 min and held at this temperature for 10 min, and then increased to 300 °C in 30 min and held at this final temperature for 5 min. The target PAHs were scanned and detected by an MSD (5975C, Agilent Technology) in the selective ion monitoring (SIM) mode. Calibration curves were established by plotting the peak area ratios (analytes/injection internal standard) versus the amounts of the analytes per injection. Target PAHs were identified and quantified using comparison of retention times and chromatographic peaks with those of known calibration curves.

Text S3

For the PAHs analyses, the calibration curves were established by plotting the peak area ratios (analytes/injection internal standard) versus the amounts of the analytes per injection. Target PAHs were identified and quantified using comparison of retention times and chromatographic peaks with those of known calibration curves. Five-point calibration plots achieved linear correlation coefficients $r^2 > 0.995$. The recoveries were also determined by spiking an equivalent loading of a mid-level concentration of the calibration standard onto the sampling medium with the two internal standards (IS) of phenanthrene (phe-d_{10}) and chrysene (chr-d_{12}). Five replicate tests were conducted for each sampling media. The concentrations reported here have been corrected for their individual recoveries.

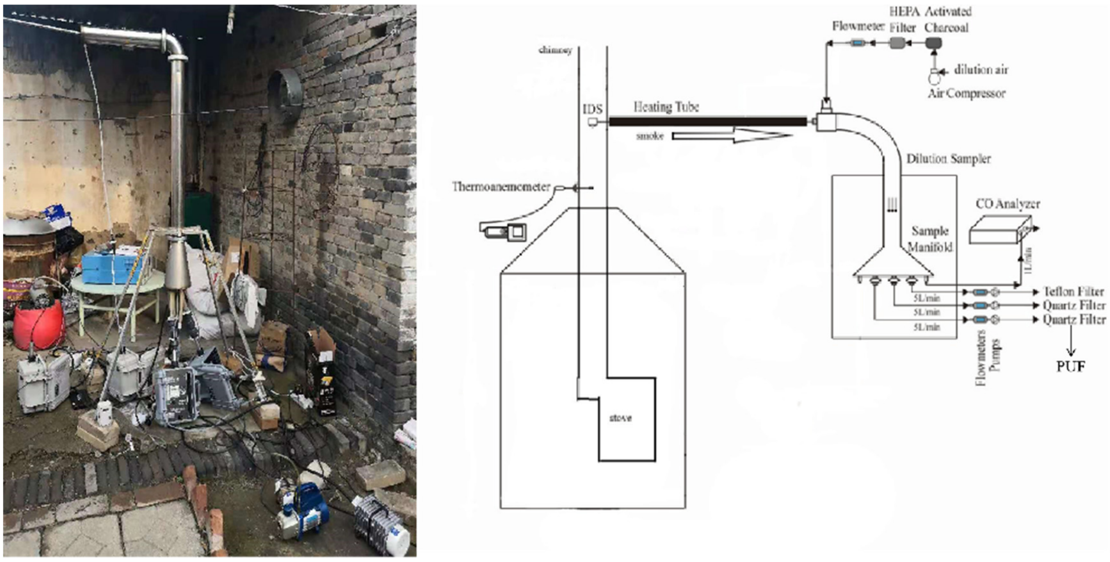

Figure S1. The simple structure of dilution system.

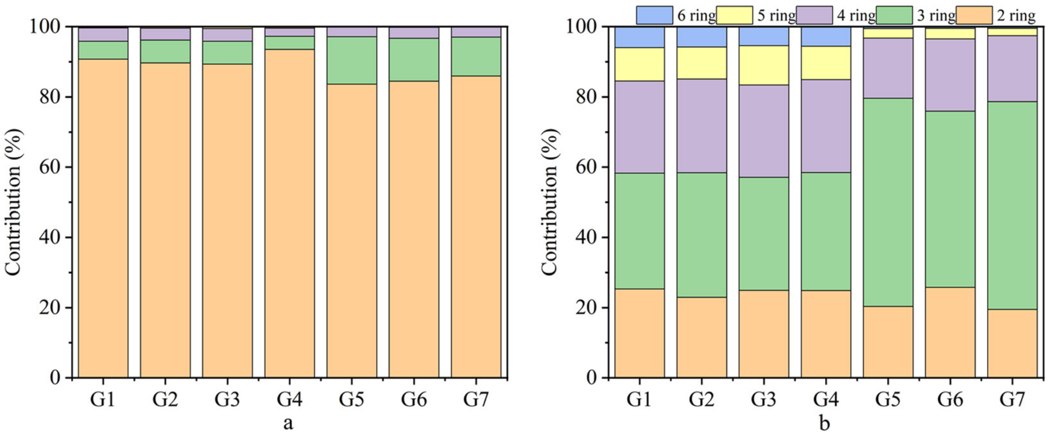

Figure S2. The number of aromatic rings distributions of PAHs in gaseous (**a**) and particulate phases (**b**).

Table S1. Individual profile of PAHs (MW = molar weight, LOD = limit of detection) (Mean ± Standard Deviation).

Particulate phase	Abbreviation	MW (g/mol)	rings	LOD (µg)
2-methylnaphthalene	2M-NAP	142.2	2	0.00623
acenaphthylene	ACY	152.2	2	0.00435
acenaphthene	ACE	154.21	2	0.00315
fluorene	FLO	166.22	2	0.00460
phenanthrene	PHE	178.23	3	0.00590
anthracene	ANT	178.23	3	0.01020
pyrene	PYR	202.25	4	0.00950
fluoranthene	FLA	202.26	4	0.00820
cyclopenta[cd]pyrene	CPP	226.27	4	0.00108
benzo[a]anthracene	BaA	228.29	4	0.01030
chrysene	CHR	228.29	4	0.00820
benzo[b]fluoranthene	BbF	252.31	4	0.00420
benzo[k]fluoranthene	BkF	252.31	4	0.00398
benzo[e]pyrene	BeP	252.31	5	0.00626
benzo[a]pyrene	BaP	252.31	5	0.00356
perylene	PER	252.31	5	0.00038
indeno[1,2,3-cd]pyrene	IcdP	276.33	5	0.00623
benzo[ghi]perylene	BghiP	276.33	6	0.00365
dibenzo[a,h]anthracene	DBahA	278.35	5	0.00099
dibenzo[a,e]pyrene	DBaeP	302.37	6	0.00359
6H-benzo(c,d)pyrene-6-one	BcdPQ	254.28	5	0.00850
5-nitroacenaphthene	5N-ACE	199.2	2	0.00650
2-nitrofluorene	2N-FLO	211.22	2	0.00490
9-nitroanthracene	9N-ANT	223.23	3	0.00820
3-nitrofluoranthene	3N-FLA	247.25	3	0.01060
1-nitropyrene	1N-PYR	247.26	4	0.01220
6-nitrochrysene	6N-CHR	273	4	0.01020
1,3-dinitropyrene	1,3-DNP	292.26	4	0.00980
1,6-dinitropyrene	1,6-DNP	292.26	4	0.01560

Table S2. The quantified ion and extraction recovery for each PAHs (SD = Standard Deviation).

PAH		Particle phase		Gas Phase	
**PAH/OPAH/APAH**	**Qual Ion**	**Recovery-Mean**	**Recovery-SD**	**Recovery-Mean**	**Recovery-SD**
acenaphthylene	152	78.6%	6.2%	69.2%	7.7%
acenaphthene	154	81.9%	8.7%	71.1%	6.8%
fluorene	166	86.1%	5.9%	80.8%	7.0%
phenanthrene	178	90.0%	4.8%	82.5%	5.5%
anthracene	178	90.0%	5.1%	88.3%	5.8%
fluoranthene	202	90.4%	5.3%	89.3%	6.0%
pyrene	202	91.0%	7.5%	88.2%	6.2%
benzo[a]anthracene	228	92.2%	4.8%	90.0%	5.1%
chrysene	228	93.3%	4.6%	91.0%	5.0%
benzo[b]fluoranthene	252	93.4%	5.8%	91.7%	6.2%
benzo[k]fluoranthene	252	93.4%	4.9%	94.6%	5.2%
benzo[e]pyrene	252	93.8%	6.0%	94.3%	6.3%
benzo[a]pyrene	252	95.8%	5.3%	95.3%	5.5%
indeno[1,2,3-cd]pyrene	276	95.0%	5.3%	94.2%	5.4%
dibenzo[a,h]anthracene	278	95.0%	5.6%	94.0%	5.1%
benzo[ghi]perylene	276	95.2%	4.1%	93.1%	4.5%
dibenzo[a,e]pyrene	302	95.5%	4.9%	92.0%	4.9%
2-methylnaphthalene	142	82.7%	4.2%	80.0%	6.7%
cyclopenta[cd]pyrene	226	90.5%	4.5%	90.1%	4.5%
6H-benzo(c,d)pyrene-6-one	254	93.6%	5.5%	93.1%	5.1%
**PAH**		**Particle Phase**		**Gas Phase**	
**NPAHs**	**Qual Ion**	**Recovey-Mean**	**Recovery-SD**	**Recovery-Mean**	**Recovery-SD**
5-nitroacenaphthene	199	91.8%	5.2%	89.5%	4.9%
2-nitrofluorene	211	92.1%	5.1%	88.7%	4.8%
9-nitroanthracene	223	94.2%	4.8%	90.0%	4.0%
3-nitrofluoranthene	247	95.1%	4.0%	94.4%	4.8%
1-nitropyrene	247	95.8%	5.8%	95.7%	5.4%
6-nitrochrysene	273	96.3%	5.0%	96.5%	5.7%
1,3-dinitropyrene	292	97.5%	5.1%	94.7%	4.8%
1,6-dinitropyrene	292	98.6%	4.3%	94.0%	4.8%

Table S3. TEF of 29 PAHs involved in this study.

PAH Species	TEF	MEF
Naphthalane	0.001	-
Acenaphthylene	0.001	-
Acenapthene	0.001	0.00056
Fluorene	0.001	-
Phenanthrene	0.001	-
Anthracene	0.01	-
Fluoranthene	0.001	-
Pyrene	0.001	-
benzo[a]anthracene	0.1	0.082
Chrysene	0.01	0.017
benzo[b]fluoranthene	0.1	0.25
benzo[k]fluoranthene	0.1	0.11
benzo[e]pyrene	0.002	-
benzo[a]pyrene	1	1
indeno[1,2,3-cd]pyrene	0.1	0.31
dibenzo[a,h]anthracene	5	0.29
benzo[ghi]perylene	0.01	0.19
dibenzo[a,e]pyrene	2.9	-
2-methylnaphthalene	0.001	-
cyclopenta[cd]pyrene	6.9	-
6H-benzo(c,d)pyrene-6-one	0.32	-
5-nitroacenaphthene	0.01	-
2-nitrofluorene	0.01	-
9-nitroanthracene	0.0032	-
3-nitrofluoranthene	0.0026	-
1-nitropyrene	0.1	-
6-nitrochrysene	10	-
1,3-dinitropyrene	0.031	-
1,6-dinitropyrene	0.28	-

Table S4. Parameters settings in non-cancer and cancer risk assessment.

Parameter	Adult Male	Adult Female
Inhalation rate	$0.8\ m^3{\cdot}h^{-1}$	$0.59\ m^3{\cdot}h^{-1}$
Average BW [a]	67.1 kg	57.3 kg
Average time spend in indoor	8 hr·day^{-1} [b]	8 hr·day^{-1}
Annual heating period	120 days·year^{-1} [c]	120 days·year^{-1}
Total number of exposure days	15695 days	15695 days
Average lifetime	70 years [d]	70 years

[a] National Statistics Bureau of China;.

[b] ~7 hours for sleeping and ~3 hours for resting and relaxing. Due to the habit of the residents, the bedrooms undertook the function of general bedrooms and living rooms, thus 10 hours were all spent in bedrooms in calculation;

[c] Heating period generally from 1st Dec. to 10th Mar next year, totally ~100 days per year;

[d] According to the risk model by USEPA.

Table S5. BaP_{MEQ} (MEQ) of gas and particulate phases PAHs from seven different combustion scenarios.

	G1	G2	G3	G4	G5	G6	G7
Gaseous phase PAHs	0.03	0.03	1.12	2.85	0.31	0.59	0.21
Particulate phase PAHs	0.29	0.29	10.66	18.12	2.18	3.29	1.45

Table S6. Cancer risk for Males of individual PAHs species in gaseous phase.

	Species	G1	G2	G3	G4	G5	G6	G7
pPAHs	NAP	1.55E-05	1.72E-05	4.00E-04	8.98E-04	2.71E-04	4.58E-04	2.57E-04
	ACY	1.58E-08	1.85E-08	5.62E-07	8.03E-07	1.35E-05	2.87E-05	1.26E-05
	ACE	1.62E-08	1.60E-08	7.72E-07	8.84E-07	8.64E-07	1.42E-06	6.67E-07
	FLO	3.74E-07	3.14E-07	6.94E-06	1.19E-05	3.89E-06	5.30E-06	1.56E-06
	PHE	7.23E-07	1.11E-06	2.54E-05	2.80E-05	3.18E-05	4.28E-05	2.25E-05
	ANT	1.21E-06	1.24E-06	2.90E-05	4.78E-05	3.75E-05	6.32E-05	1.51E-05
	FLA	9.28E-08	7.97E-08	3.24E-06	3.81E-06	1.27E-05	2.38E-05	1.07E-05
	PYR	1.46E-07	1.61E-07	3.50E-06	4.37E-06	6.01E-06	1.29E-05	5.50E-06
	BaA	1.05E-05	1.69E-05	3.02E-04	4.79E-04	1.29E-04	2.05E-04	1.14E-04
	CHR	3.54E-06	2.74E-06	7.23E-05	1.02E-04	1.08E-05	1.98E-05	1.83E-05
	BbF	8.73E-06	7.49E-06	3.12E-04	4.11E-04	3.21E-05	4.61E-05	1.90E-05
	BkF	7.88E-07	6.55E-07	3.07E-05	3.25E-05	5.38E-05	8.45E-05	2.28E-05
	BeP	7.71E-08	9.43E-08	2.24E-06	2.25E-06	3.02E-07	9.42E-07	3.64E-07
	BaP	3.70E-05	3.76E-05	1.14E-03	2.71E-03	1.99E-04	5.29E-04	1.04E-04
	CPP	2.03E-05	3.15E-05	7.40E-04	1.39E-03	2.46E-03	2.96E-03	1.46E-03
aPAHs	2M-NAP	8.46E-07	7.19E-07	1.94E-05	3.36E-05	2.06E-06	1.22E-05	2.13E-06
oPAHs	BcdPQ	2.37E-06	2.18E-06	1.19E-04	1.01E-04	3.53E-05	9.55E-05	1.73E-05
nPAHs	5N-ACE	4.80E-09	4.17E-09	1.23E-07	1.58E-07	3.65E-08	7.75E-08	3.81E-08
	2N-FLO	1.74E-09	1.43E-09	7.17E-08	6.22E-08	1.73E-08	2.98E-08	1.12E-08
	9N-PHE	1.88E-10	1.77E-10	7.19E-09	6.40E-09	4.92E-09	5.43E-09	3.39E-09
	3N-FLA	3.28E-10	4.41E-10	1.20E-08	1.40E-08	3.24E-09	8.40E-09	4.10E-09
	1N-PYR	1.04E-07	6.14E-08	2.06E-06	2.56E-06	1.64E-06	2.32E-06	7.44E-07
	6N-CHR	2.44E-07	3.49E-07	1.12E-05	1.18E-05	8.58E-05	1.43E-04	4.20E-05
	1,3-DNP	2.15E-09	2.95E-09	9.08E-08	1.39E-07	1.74E-07	2.72E-07	1.24E-07
	1,6-DNP	1.67E-08	2.29E-08	4.68E-07	1.41E-06	2.79E-06	5.26E-06	1.65E-06

Table S7. Cancer risk for Females of individual PAHs species in gaseous phase.

	Species	G1	G2	G3	G4	G5	G6	G7
pPAHs	NAP	1.34E-05	1.48E-05	3.45E-04	7.76E-04	2.34E-04	3.96E-04	2.22E-04
	ACY	1.36E-08	1.60E-08	4.85E-07	6.93E-07	1.17E-05	2.48E-05	1.09E-05
	ACE	1.40E-08	1.38E-08	6.66E-07	7.64E-07	7.46E-07	1.22E-06	5.76E-07
	FLO	3.23E-07	2.71E-07	5.99E-06	1.02E-05	3.36E-06	4.58E-06	1.35E-06
	PHE	6.24E-07	9.58E-07	2.20E-05	2.42E-05	2.74E-05	3.69E-05	1.94E-05
	ANT	1.05E-06	1.07E-06	2.50E-05	4.13E-05	3.24E-05	5.46E-05	1.30E-05
	FLA	8.02E-08	6.88E-08	2.80E-06	3.29E-06	1.10E-05	2.05E-05	9.25E-06
	PYR	1.26E-07	1.39E-07	3.03E-06	3.77E-06	5.19E-06	1.11E-05	4.75E-06
	BaA	9.08E-06	1.46E-05	2.61E-04	4.14E-04	1.11E-04	1.77E-04	9.84E-05
	CHR	3.05E-06	2.37E-06	6.24E-05	8.84E-05	9.32E-06	1.71E-05	1.58E-05
	BbF	7.54E-06	6.47E-06	2.69E-04	3.55E-04	2.78E-05	3.98E-05	1.64E-05
	BkF	6.81E-07	5.66E-07	2.65E-05	2.81E-05	4.65E-05	7.30E-05	1.97E-05
	BeP	6.66E-08	8.15E-08	1.94E-06	1.94E-06	2.61E-07	8.13E-07	3.14E-07
	BaP	3.19E-05	3.25E-05	9.87E-04	2.34E-03	1.72E-04	4.57E-04	8.94E-05
	CPP	1.75E-05	2.72E-05	6.39E-04	1.20E-03	2.12E-03	2.55E-03	1.26E-03
aPAHs	2M-NAP	7.31E-07	6.21E-07	1.68E-05	2.91E-05	1.78E-06	1.05E-05	1.84E-06
oPAHs	BcdPQ	2.04E-06	1.88E-06	1.02E-04	8.68E-05	3.05E-05	8.25E-05	1.50E-05
nPAHs	5N-ACE	4.15E-09	3.60E-09	1.06E-07	1.37E-07	3.16E-08	6.69E-08	3.29E-08
	2N-FLO	1.50E-09	1.24E-09	6.19E-08	5.37E-08	1.49E-08	2.58E-08	9.64E-09
	9N-PHE	1.62E-10	1.53E-10	6.21E-09	5.53E-09	4.25E-09	4.69E-09	2.92E-09
	3N-FLA	2.83E-10	3.81E-10	1.04E-08	1.21E-08	2.80E-09	7.25E-09	3.54E-09
	1N-PYR	9.02E-08	5.30E-08	1.78E-06	2.21E-06	1.42E-06	2.00E-06	6.42E-07
	6N-CHR	2.11E-07	3.01E-07	9.70E-06	1.02E-05	7.41E-05	1.24E-04	3.63E-05
	1,3-DNP	1.85E-09	2.55E-09	7.84E-08	1.20E-07	1.50E-07	2.35E-07	1.07E-07
	1,6-DNP	1.45E-08	1.98E-08	4.05E-07	1.22E-06	2.41E-06	4.54E-06	1.43E-06

Table S8. Cancer risk for Males of individual PAHs species in particulate phase.

	Species	G1	G2	G3	G4	G5	G6	G7
pPAHs	ACY	3.44E-08	2.94E-08	9.95E-07	1.44E-06	1.88E-05	4.59E-05	1.95E-05
	ACE	3.15E-08	2.84E-08	1.05E-06	1.09E-06	1.43E-06	2.24E-06	1.13E-06
	FLO	6.44E-07	7.25E-07	1.66E-05	2.93E-05	6.39E-06	1.07E-05	3.59E-06
	PHE	1.63E-06	1.89E-06	4.51E-05	7.22E-05	4.70E-05	6.74E-05	3.63E-05
	ANT	2.97E-06	2.83E-06	7.35E-05	1.14E-04	8.91E-05	1.68E-04	5.65E-05
	FLA	2.72E-07	2.44E-07	7.35E-06	8.68E-06	3.12E-05	5.68E-05	3.72E-05
	PYR	3.88E-07	3.74E-07	9.13E-06	1.54E-05	1.47E-05	4.00E-05	1.58E-05
	BaA	2.85E-05	3.15E-05	8.34E-04	1.34E-03	3.43E-04	5.04E-04	2.70E-04
	CHR	6.23E-06	6.88E-06	1.72E-04	2.38E-04	2.83E-05	5.23E-05	4.10E-05
	BbF	4.06E-05	3.93E-05	1.23E-03	1.90E-03	1.32E-04	2.42E-04	8.45E-05
	BkF	4.67E-06	3.97E-06	1.28E-04	1.72E-04	2.05E-04	3.80E-04	1.30E-04
	BeP	3.35E-07	3.72E-07	1.36E-05	1.06E-05	1.74E-06	4.56E-06	1.88E-06
	BaP	2.66E-04	2.25E-04	8.74E-03	1.33E-02	1.29E-03	2.73E-03	6.21E-04
	IcdP	7.68E-06	7.33E-06	1.77E-04	2.98E-04	8.46E-05	1.56E-04	7.62E-05
	DBahA	3.45E-04	4.23E-04	9.95E-03	1.46E-02	6.94E-04	7.68E-04	4.23E-04
	BghiP	2.69E-06	2.95E-06	7.19E-05	1.08E-04	7.90E-06	1.37E-05	6.44E-06
	DBaeP	3.83E-04	2.93E-04	8.06E-03	1.35E-02	8.16E-05	1.79E-04	1.12E-04
	CPP	3.85E-05	6.62E-05	1.87E-03	3.09E-03	5.43E-03	6.85E-03	3.09E-03
aPAHs	2M-NAP	9.87E-07	7.79E-07	2.65E-05	3.70E-05	2.78E-06	1.46E-05	2.11E-06
oPAHs	BcdPQ	1.56E-05	1.53E-05	4.64E-04	6.39E-04	2.25E-04	5.26E-04	1.05E-04
nPAHs	5N-ACE	8.02E-09	7.08E-09	1.98E-07	2.84E-07	4.75E-08	1.24E-07	4.57E-08
	2N-FLO	2.29E-09	2.51E-09	7.30E-08	1.06E-07	2.10E-08	3.22E-08	1.11E-08
	9N-PHE	3.18E-10	2.46E-10	7.85E-09	1.12E-08	7.16E-09	1.26E-08	5.87E-09
	3N-FLA	6.37E-10	8.62E-10	2.30E-08	3.04E-08	5.54E-09	1.85E-08	7.69E-09
	1N-PYR	1.29E-07	1.07E-07	3.25E-06	5.43E-06	2.59E-06	4.61E-06	1.54E-06
	6N-CHR	7.11E-07	9.48E-07	2.11E-05	3.45E-05	2.09E-04	3.44E-04	1.20E-04
	1,3-DNP	7.06E-09	7.05E-09	2.03E-07	2.62E-07	3.32E-07	5.48E-07	2.76E-07
	1,6-DNP	5.44E-08	5.10E-08	1.47E-06	3.00E-06	7.87E-06	1.33E-05	3.69E-06

Table S9. Cancer risk for Females of individual PAHs species in particulate phase.

	**Species**	**G1**	**G2**	**G3**	**G4**	**G5**	**G6**	**G7**
pPAHs	ACY	2.97E-08	2.54E-08	8.59E-07	1.24E-06	1.63E-05	3.97E-05	1.69E-05
	ACE	2.72E-08	2.45E-08	9.03E-07	9.40E-07	1.23E-06	1.93E-06	9.77E-07
	FLO	5.56E-07	6.26E-07	1.43E-05	2.53E-05	5.52E-06	9.24E-06	3.10E-06
	PHE	1.40E-06	1.63E-06	3.90E-05	6.24E-05	4.06E-05	5.82E-05	3.13E-05
	ANT	2.56E-06	2.44E-06	6.35E-05	9.85E-05	7.69E-05	1.45E-04	4.88E-05
	FLA	2.35E-07	2.11E-07	6.35E-06	7.49E-06	2.70E-05	4.91E-05	3.21E-05
	PYR	3.35E-07	3.23E-07	7.89E-06	1.33E-05	1.27E-05	3.45E-05	1.36E-05
	BaA	2.46E-05	2.72E-05	7.20E-04	1.16E-03	2.96E-04	4.35E-04	2.33E-04
	CHR	5.38E-06	5.94E-06	1.49E-04	2.05E-04	2.45E-05	4.52E-05	3.54E-05
	BbF	3.51E-05	3.39E-05	1.06E-03	1.64E-03	1.14E-04	2.09E-04	7.30E-05
	BkF	4.03E-06	3.43E-06	1.11E-04	1.48E-04	1.77E-04	3.28E-04	1.12E-04
	BeP	2.90E-07	3.21E-07	1.17E-05	9.19E-06	1.50E-06	3.94E-06	1.62E-06
	BaP	2.30E-04	1.94E-04	7.55E-03	1.15E-02	1.11E-03	2.36E-03	5.37E-04
	IcdP	6.63E-06	6.33E-06	1.53E-04	2.57E-04	7.30E-05	1.35E-04	6.58E-05
	DBahA	2.98E-04	3.65E-04	8.59E-03	1.27E-02	6.00E-04	6.63E-04	3.66E-04
	BghiP	2.32E-06	2.54E-06	6.21E-05	9.36E-05	6.82E-06	1.18E-05	5.56E-06
	DBaeP	3.31E-04	2.53E-04	6.96E-03	1.16E-02	7.05E-05	1.55E-04	9.68E-05
	CPP	3.32E-05	5.72E-05	1.62E-03	2.67E-03	4.69E-03	5.92E-03	2.67E-03
aPAHs	2M-NAP	8.52E-07	6.72E-07	2.29E-05	3.19E-05	2.40E-06	1.26E-05	1.82E-06
oPAHs	BcdPQ	1.35E-05	1.32E-05	4.00E-04	5.52E-04	1.94E-04	4.55E-04	9.05E-05
nPAHs	5N-ACE	6.92E-09	6.12E-09	1.71E-07	2.46E-07	4.10E-08	1.07E-07	3.94E-08
	2N-FLO	1.98E-09	2.16E-09	6.31E-08	9.12E-08	1.82E-08	2.78E-08	9.59E-09
	9N-PHE	2.74E-10	2.13E-10	6.78E-09	9.68E-09	6.18E-09	1.08E-08	5.07E-09
	3N-FLA	5.50E-10	7.45E-10	1.98E-08	2.62E-08	4.78E-09	1.59E-08	6.65E-09
	1N-PYR	1.11E-07	9.20E-08	2.81E-06	4.69E-06	2.24E-06	3.98E-06	1.33E-06
	6N-CHR	6.14E-07	8.18E-07	1.82E-05	2.98E-05	1.81E-04	2.97E-04	1.04E-04
	1,3-DNP	6.09E-09	6.09E-09	1.76E-07	2.27E-07	2.86E-07	4.73E-07	2.38E-07
	1,6-DNP	4.70E-08	4.40E-08	1.27E-06	2.59E-06	6.80E-06	1.15E-05	3.18E-06
